# HIV-related stigma among Spanish-speaking Latinos in an emerging immigrant city following the *Solo Se Vive Una Vez* social marketing campaign

**DOI:** 10.1371/journal.pone.0274888

**Published:** 2022-10-06

**Authors:** Suzanne M. Dolwick Grieb, Matthew Velez, Edward W. Corty, Ronald E. Saxton, Alejandra Flores-Miller, Harita S. Shah, Kathleen R. Page

**Affiliations:** 1 Center for Child and Community Health Research, Johns Hopkins University School of Medicine, Baltimore, Maryland, United States of America; 2 Feinberg School of Medicine, Northwestern University, Chicago, Illinois, United States of America; 3 Johns Hopkins Bloomberg School of Public Health, Baltimore, Maryland, United States of America; 4 Division of Infectious Diseases, Johns Hopkins University School of Medicine, Baltimore, Maryland, United States of America; 5 Department of Medicine, University of Chicago, Chicago, Illinois, United States of America; PLOS ONE, UNITED KINGDOM

## Abstract

HIV-related stigma exacerbates Latino immigrants’ risk of HIV infection and delayed care. Following the implementation of the social marketing campaign *Sólo Se Vive Una Vez* (You Only Live Once) to increase HIV testing that addressed stigmatizing beliefs, we conducted a survey among Latinos in Baltimore, Maryland (N = 357). The aims of this paper are to 1) characterize the sociodemographic characteristics, HIV-related stigma beliefs, and testing behaviors of the survey respondents by campaign exposure, and 2) model the effects of *Vive* exposure on stigma beliefs and testing behaviors. Comparing post-campaign survey respondents exposed and unexposed to the campaign to survey findings previously obtained and reported before the campaign implementation, respondents to the post-*Vive* survey continued to hold high levels of stigma beliefs, and compared to the pre-*Vive* survey sample, were more likely to hold four or more stigmatizing beliefs (from the six survey items). Among the post-*Vive* survey respondents, those for whom religion was important or very important had an increased odds of 1.6 of holding four or more stigmatizing beliefs. Survey respondents who were exposed to the campaign, however, had an increased odds of 2.25 of reporting ever having been tested for HIV. Our findings demonstrate the importance of the changing social context in addressing stigma within emerging immigrant communities and highlight the critical role of religious leaders in efforts to address HIV-related stigma.

## Introduction

HIV/AIDS disproportionately affects Latinos in the United States (U.S.) [1, 2], and despite substantial progress made in the U.S. to reduce HIV incidence, Centers for Disease Control and Prevention (CDC) estimates suggest new infections continue to rise among Latinos. Between 2010 and 2016, HIV incidence in the U.S. increased by 14% among Latinos, while it decreased 6% overall [3]. Moreover, Latinos are more likely to report never having been offered an HIV test [4], are less likely to be aware of their HIV status [3], and present to healthcare services with more advanced disease than their non-Hispanic counterparts [5–7].

Latino immigrants account for a third of all HIV diagnoses among Latinos [8] and are at greater risk than their U.S.-born peers for delayed diagnosis and presentation to care [9, 10]. Latino immigrants face numerous challenges in the U.S. that exacerbate their vulnerability to HIV infection and limit their access to healthcare services [11–14]. Importantly, HIV-related stigma has been associated with reluctance to be HIV tested among Latinos and produces a barrier to HIV education and linkage to care [6, 12, 15, 16]. Stigma is a powerful discrediting social label that dramatically impacts how individuals view themselves and are viewed by others. Cultural meanings given to stigmatized attributes, such as HIV infection, are reproduced through social interactions and are therefore linked to the actions of groups of people within specific contexts [17, 18]. Among Latinos in Baltimore surveyed in 2014–2015, for example, half of respondents agreed that people living with or thought to be living with HIV lose respect of standing, and three-quarters agreed that people get HIV because they engage in irresponsible behavior [13]. This stigmatizing association of HIV with “irresponsible behavior” is often tied to religious taboos on homosexuality, multiple sex partners, and drug use [19, 20].

HIV-related stigma reduction is a priority for reducing HIV disparities within the national HIV/AIDS prevention strategy as well as by the national Hispanic/Latinx Delegation on HIV/AIDS [21, 22]. Specifically, Guilamo-Ramos and colleagues [22] argue that stigma-reduction campaigns are needed to decrease HIV-related stigma in Latino communities at large, and not only through targeting at-risk or HIV-positive individuals.

Social marketing has been shown to be an effective strategy for promoting HIV screening and reducing HIV stigma [23, 24]. To improve HIV testing and linkage to care among Latinos in Baltimore, a new immigrant receiving community with a rapidly growing immigrant population, we developed the multilevel social marketing campaign *Sólo Se Vive Una Vez* (You Only Live Once, henceforth referred to as *Vive*) through partnerships with the Baltimore City Health Department (BCHD) Latino outreach team, community members and leaders, Altavista Studios, Maryland Institute College of Art (MICA), and academic investigators from Johns Hopkins University [25–28]. The individual-level component of the campaign was a website (www.solovive.org) with culturally sensitive video modules which individuals could select based on their perceived barrier(s) to HIV testing and/or HIV-related stigmatizing beliefs. The community-level components of the campaign included advertisements on multiple mediums (e.g., social media, dating applications used by men who have sex with men, local Spanish-language radio stations, billboards, buses, newspapers, and posters) as well as community outreach events.

Within the individual- and community-level components, *Vive* featured messaging that addressed the most common stigmatizing beliefs and barriers to testing, as guided by prior research. These barriers were (translated from Spanish): I am in a stable relationship, I don’t have legal documentation to be in the U.S., I feel fine/healthy, I am scared of the result, I am not at risk for HIV, I don’t have health insurance, I am afraid of needles, I don’t have time, I am ashamed/embarrassed, and I don’t have sex with just anybody [12, 13, 26]. Videos and corresponding advertisements (e.g., social media ads, posters) also included the motivational messages “Saber es poder” (Knowledge is power) and “El VIH solo es peligroso cuando lo ignoras” (HIV only is dangerous when you ignore it) as well as information about Spanish-language services at the health department and contact information for Latino outreach workers.

The *Sólo Se Vive Una Vez* campaign was implemented in Baltimore between June 22, 2018 and January 12, 2019. The aims of this paper are to 1) characterize the sociodemographic characteristics, HIV-related stigma beliefs, and testing behaviors of the survey respondents by campaign exposure, and 2) model the effects of *Vive* exposure on stigma beliefs and testing behaviors. Findings on the campaign’s reach and broader impact on HIV screening among Latinos in Baltimore are published elsewhere [27, 28].

## Methods

### Study design

We conducted a cross sectional study between September and December 2019 to characterize HIV-related stigma among a convenience sample of Spanish-speaking Latino/Latina adults in Baltimore, Maryland and to assess exposure to the *Vive* campaign. The same survey, utilizing the same methodology, was also conducted within this community prior to the *Vive* campaign launch (September 2014- August 2015) [13].

The study design is described in detail elsewhere [13], but briefly, trained research assistants recruited respondents at street- and community-based venues frequented by foreign-born Latinos, as informed by our prior work mapping and assessing locations for sampling the Latino immigrant population in Baltimore [29]. Participants were eligible for inclusion if they were over 18 years of age, self-identified as Latino/Latina, and were able to complete the survey in Spanish. Since the goal was to explore HIV-related stigma in the local community of Latino immigrants, U.S. born Latinos interacting with Latino immigrants in these spaces were also able to participate if they met the eligibility requirements. Survey data was collected in Spanish on a tablet using Qualtrics formatted with audio. The survey included a statement of consent and took approximately 5–10 minutes to complete. Participants received $5 compensation upon completion of the survey. The Johns Hopkins School of Medicine Institutional Review Board (IRB) approved the study.

### Measures

The survey included questions regarding socio-demographic characteristics and HIV test history, as well as six items to assess HIV-related stigma. Sociodemographic characteristics collected included age, gender, country of origin, education, religious affiliation, importance of religion, time in the U.S., as well as differences in HIV testing history (ever being tested for HIV, and time since last test if ever tested), and knowing someone infected with or thought to be infected with HIV. To assess HIV-related stigma, we utilized validated items recommended by the UNAIDS-funded HIV Stigma Network, selected to incorporate multiple stigma domains: fear of transmission and disease, anticipated stigma, association with shame, blame and judgment, and support for discriminatory actions or policies against people living with HIV/AIDS (PLHA) [30]. The latter three domains address personal attitudes as well as perceived community responses towards PLHA. The items and domains were selected to allow differentiation between specific drivers and causes of HIV stigma, such as fear of infection due to lack of knowledge about transmission and moralistic attitudes and beliefs surrounding PLHA. Response choices for each stigma item included “Agree” and “Disagree,” or “Yes,” “No,” and “Depends,” depending on the phrasing of the item (as a statement or question). All items also had the response choices “I don’t know/No opinion” and “I prefer not to respond.” The *Vive* campaign was informed by our formative research findings, including the initial pre-*Vive* survey findings. As a result, the *Vive* campaign did not address each stigma item in this survey directly; however, these stigma measures were repeated in the post-*Vive* survey for comparison to the pre-*Vive* survey findings. These repeated measures may provide insight into the effectiveness of new ideas and information communicated through the *Vive* campaign, as education is understood to be an important component of stigma reduction [31].

To assess exposure to the *Vive* campaign, participants were asked: “Have you previously seen or heard of the *¡Solo Se Vive Una Vez*! campaign?” Participants who answered “Yes” were then asked if they considered testing, or did get tested, for HIV because of seeing the campaign. They were also asked to report all the ways they saw or heard about the campaign from a list of possible sources (i.e., website, Facebook, billboard, poster, radio).

### Analysis

Guided by the literature [16, 19, 20, 32] and our previous research [13], sociodemographic variables were dichotomized as follows. Education was dichotomized as completing secondary school or higher versus not having completed secondary school. Importance of religion was dichotomized between important or very important versus somewhat important or not important. Time in the U.S. was dichotomized as 5 or more years versus less than 5 years. Time since last test was dichotomized as within the past year versus over one year ago (never, over one year ago, or could not remember when).

Self-reported exposure to the campaign was used to categorize three groups of survey respondents: the pre-*Vive* group consisting of those surveyed prior to development and implementation of the campaign [13]; the post-*Vive-*unexposed group surveyed after the campaign was implemented, but who did not self-report seeing or hearing of the campaign; and the post-*Vive*-exposed group surveyed after the campaign was implemented and who self-reported exposure.

### Aim 1: Characterize the sociodemographic characteristics, HIV-related stigma beliefs, and testing behaviors of the survey respondents by campaign exposure

We assessed possible differences in sociodemographic characteristics thought to influence HIV-related stigma beliefs and testing behaviors between the three cohort/exposure groups–pre-*Vive* (n = 312), unexposed post-*Vive* (n = 218) and exposed post-*Vive* (n = 139)–using a one-way ANOVA to test for significant differences in continuous age distributions, and Pearson’s chi-squared tests for all categorical variables.

We also assessed possible differences in the percent of respondents in agreement with HIV-related stigma beliefs and self-reported testing behaviors between the three cohort/exposure groups. Pearson’s chi-squared tests were used to test for statistical significance of these differences in agreement with each stigma item between the samples. Chronbach’s alpha was calculated on the six stigma items to test internal consistency. Chronbach’s alpha was 0.50, indicating low internal consistency; thus, each stigma item was analyzed individually.

### Aim 2: Model effects of *Vive* exposure on HIV-related stigma beliefs and HIV testing behaviors

To test the hypothesis that *Vive* campaign exposure would reduce agreement with HIV-related stigma and increase HIV testing behaviors, we constructed logistic regression models with the pre-*Vive* category as the reference group and compared the effects non-exposure and exposure on stigma and testing outcomes. Adjusted logistic regression models controlled for factors observed to differ significantly between the groups (education level, importance of religion, and time spent in the U.S.) as well as for factors typically associated with stigma from the literature (gender, age, and knowing PLHA). None of the adjusted models violated the Hosmer-Lemeshow goodness-of-fit test. All models and analyses were conducted using STATA Software Version 16.0.

## Results

### Aim 1: Characterize the sociodemographic characteristics, HIV-related stigma beliefs, and testing behaviors of the survey respondents by campaign exposure

The post-*Vive* survey was completed by 357 members of the Latino community; 162 (45.4%) identified as male and 195 (54.6%) as female, with a mean age of 35.7 (SD = 11.4) and 35.2 (SD = 10.5) years, respectively. The majority reported their country of origin as Mexico (30.5%), Honduras (23.0%), or El Salvador (19.6%); few participants were born in the U.S. mainland (6.4%). The majority surveyed had not completed a secondary school education (52.1%), reported that their religion was important or very important to them (67.2%), and had lived in the U.S. for over 5 years (61.9%). No significant socio-demographic differences were found between the male and female participants.

Over one-third (n = 139, 38.9%) of post-*Vive* survey respondents reported exposure to the *Vive* campaign; 34.6% of males and 42.6% of females reported seeing or hearing about the campaign. Community events were identified as the most common way respondents learned about *Vive* (39.6%), followed by Facebook (35.3%). Considering the samples of pre-*Vive*, post-*Vive*-unexposed, and post-*Vive*-exposed, statistically significant differences were observed for education level, importance of religion, and time lived in the U.S. (Table 1).

**Table 1:** Socio-demographic characteristics of the pre-*Vive*^a^ (n = 312), post-*Vive*-unexposed (n = 218), and post-*Vive*-exposed (n = 139) respondents survey respondents.

Sociodemographic characteristic	Pre-*Vive* n (%)	Post-*Vive*- unexposed n (%)	Post-*Vive*- exposed n (%)	p-value^b^
Age (mean, SD)	35.4 (10.6)	34.5 (10.9)	36.9 (10.8)	0.107
Gender				0.297
Female	173 (55.5)	112 (51.4)	83 (59.7)	
Male	139 (44.5)	106 (48.6)	56 (40.3)	
Country of Origin				0.131
Mexico	101 (32.4)	55 (25.2)	54 (38.9)	
El Salvador	49 (15.7)	44 (20.2)	26 (18.7)	
Guatemala	26 (8.3)	17 (7.8)	8 (5.8)	
Honduras	70 (22.4)	56 (25.7)	26 (18.7)	
United States (mainland)	13 (4.2)	12 (5.5)	11 (7.9)	
Other	53 (17.0)	34 (15.6)	14 (10.1)	
Education				0.021
Did not complete secondary school	191 (61.2)	107 (49.1)	79 (56.8)	
Completed secondary school	121 (38.8)	111 (50.9)	60 (43.2)	
Religion				0.057
Catholic	193 (61.9)	106 (48.6)	81 (58.3)	
Evangelic/Protestant	60 (19.2)	59 (27.1)	29 (20.9)	
Other	32 (10.3)	21 (9.6)	15 (10.8)	
No religion	27 (8.7)	32 (14.7)	14 (10.1)	
Importance of religion				0.010
Important or very important	74 (23.7)	78 (35.8)	39 (28.1)	
Somewhat or not important	238 (76.3)	140 (64.2)	100 (71.9)	
Time in the U.S.				0.027
Less than 5 years	96 (30.8)	91 (41.7)	45 (32.4)	
5 years of more	216 (69.2)	127 (58.3)	94 (67.6)	
Know someone living with HIV				0.698
Yes	24 (7.7)	13 (6.0)	11 (7.9)	
No / unsure	288 (92.3)	205 (94.0)	128 (92.1)	

^a^ *Solo Se Vive Una Vez (Vive)* was implemented between June 22, 2018 and January 12, 2019. The pre-*Vive* survey was conducted September 2014-August 2015 to inform the development of the *Vive* campaign. The post-*Vive* survey was conducted between September and December 2019.

^b^ One-way ANOVA (continuous age) and Pearson’s chi-squared (categorical) tests for observed sociodemographic differences between *Vive* cohort/exposure groups.

Agreement with each HIV-related stigma item and self-reported HIV testing behaviors are presented for the pre-*Vive*, post-*Vive*-unexposed, and post-*Vive*-exposed cohorts in Table 2. Among respondents in all groups, over half agreed with the statements “I fear that I could contract HIV if I came into contact with the saliva of a person living with HIV,” “People living or thought to be living with HIV lose respect or standing,” and “People get HIV because they engage in irresponsible behavior.” A statistically significant difference among the three groups was observed only for the item “I would be ashamed if someone in my family had HIV.” Among post*-Vive*-unexposed respondents, 42.7% agreed to this statement compared to 41.0% of post-*Vive*-exposed and 25.9% of pre-*Vive* respondents (p < 0.001). Post-*Vive* respondents, however, were significantly more likely to endorse four or more of the six stigma items than the pre*-Vive* respondents (p = 0.036).

**Table 2:** Agreement^a^ with HIV-related stigma beliefs and self-reported testing behaviors among pre-*Vive* (n = 312), post-*Vive*-unexposed (n = 218), and post-*Vive*-exposed (n = 139) survey respondents.

	Pre-*Vive* n (%)	Post-*Vive*- unexposed n (%)	Post-*Vive*- exposed n (%)	p-value^b^
**HIV-related Stigma Item**				
I feel hesitant to take an HIV test due to fear of people’s reaction if the test result is positive for HIV	63 (21.4)	53 (23.9)	35 (25.2)	0.647
I fear that I could contract HIV if I came into contact with the saliva of a person living with HIV	177 (59.0)	121 (55.5)	87 (62.6)	0.408
I think children living with HIV should not be able to attend school with children who are HIV negative	115 (38.1)	85 (39.0)	49 (35.3)	0.769
I would be ashamed if someone in my family had HIV	75 (25.9)	93 (42.7)	57 (41.0)	<0.001
People living or thought to be living with HIV lose respect or standing	151 (50.7)	123 (56.4)	72 (51.8)	0.418
People get HIV because they engage in irresponsible behavior	220 (74.1)	172 (78.9)	116 (83.5)	0.079
Agreement with four or more stigma beliefs	69 (27.0)	78 (35.8)	53 (38.1)	0.036
**Testing Behavior**				
Ever tested for HIV	213 (68.3)	138 (63.3)	114 (82.0)	<0.001
Tested for HIV in the past 1 year^c^	109 (51.2)	52 (37.7)	65 (57.0)	0.006

^a^ Agreement with the stigma belief items were based on “Yes/Agree,” “Depends,” and “I don’t know” responses, except for the item related to children with HIV attending school with children who are HIV negative. For this item, agreement was based on the response of “No” and “I don’t know.”

^b^ Pearson’s chi-squared tests for observed differences in stigma and testing between *Vive* cohort/exposure groups

^c^ Includes only those who have ever tested for HIV

Survey respondents differed significant with respect to ever testing for HIV and testing for HIV in the past year based on exposure to the *Vive* campaign. Among post-*Vive*-exposed respondents, 82.0% self-reported ever testing for HIV, compared to 68.3% of pre-*Vive* and 63.3% of post-*Vive*-unexposed respondents (p < 0.001). Among those ever testing for HIV, 57.0% of post-*Vive*-exposed respondents reported testing in the past year, compared to 51.2% of pre-*Vive* and 37.7% of post-*Vive*-unexposed respondents (p = 0.006).

### Aim 2: Model effects of *Vive* exposure on HIV-related stigma beliefs and HIV testing behaviors

The adjusted odds ratio (AOR) modelling effects of exposure to the *Vive* campaign on agreement with stigma belief items and HIV testing behaviors are provided in Tables 3 and 4, respectively. After controlling for observed differences in education, religiosity, and time in the U.S., as well as the effects of gender, age, and knowing someone living with HIV, both post-*Vive* groups had statistically significantly greater likelihood of agreement with the statement “I would be ashamed if someone in my family had HIV” as compared to the pre-*Vive* cohort (p < .001). The increased likelihood was less pronounced, however, among the post-*Vive*-exposed group compared to the post-*Vive*-unexposed (AORs 2.09 and 2.35, respectively). Exposure to the *Vive* campaign was associated with increased likelihood of agreement that people who get HIV through engaging in irresponsible behaviors, compared to the pre-*Vive* cohort (AOR = 1.85, 95% CI (1.08, 3.15), p = 0.025). The pro-blame trend was mirrored, but not statistically significant, in the unexposed respondents (AOR = 1.32, 95% CI (0.85, 2.04), p = 0.211). Finally, both post-*Vive* groups had statistically significantly greater likelihood of agreement with four or more stigma items.

**Table 3:** Effects of cohort and *Vive*-campaign exposure on agreement with stigma beliefs.

	AOR^a^	(95% CI)	p-value
**I feel hesitant to take an HIV test due to fear of people’s reaction if the test result is positive for HIV**			
Cohort / Campaign Exposure			
Post-unexposed	1.16	(0.75, 1.78)	0.510
Post-exposed	1.29	(0.80, 2.09)	0.297
Completed secondary school	0.73	(0.49, 1.07)	0.102
Religion is important or very important	1.44	(0.93, 2.21)	0.100
Spent 5 years or longer in the U.S.	0.73	(0.48, 1.11)	0.141
Male gender	1.50	(1.04, 2.18)	**0.032**
Age (continuous)	0.99	(0.97, 1.01)	0.371
Knows someone living or thought to be living with HIV	0.48	(0.20, 1.17)	0.106
**I fear that I could contract HIV if I came into contact with the saliva of a person living with HIV**			
Cohort / Campaign Exposure			
Post-unexposed	0.95	(0.65, 1.37)	0.772
Post-exposed	1.27	(0.83, 1.95)	0.273
Completed secondary school	0.55	(0.40, 0.77)	**<0.001**
Religion is important or very important	1.65	(1.16, 2.36)	**0.006**
Spent 5 years or longer in the U.S.	1.08	(0.75, 1.56)	0.669
Male gender	2.02	(1.46, 2.81)	**<0.001**
Age (continuous)	1.00	(0.98, 1.01)	0.639
Knows someone living or thought to be living with HIV	0.70	(0.37, 1.31)	0.261
**I think children living with HIV should not be able to attend school with children who are HIV negative**			
Cohort / Campaign Exposure			
Post-unexposed	1.20	(0.83, 1.74)	0.335
Post-exposed	0.93	(0.61, 1.43)	0.737
Completed secondary school	0.56	(0.40, 0.78)	**<0.001**
Religion is important or very important	1.95	(1.34, 2.85)	**<0.001**
Spent 5 years or longer in the U.S.	1.03	(0.72, 1.49)	0.858
Male gender	1.06	(0.77, 1.46)	0.728
Age (continuous)	1.00	(0.98, 1.02)	0.899
Knows someone living or thought to be living with HIV	0.78	(0.40, 1.49)	0.443
**I would be ashamed if someone in my family had HIV**			
Cohort / Campaign Exposure			
Post-unexposed	2.33	(1.58, 3.45)	**<0.001**
Post-exposed	2.14	(1.38, 3.32)	**<0.001**
Completed secondary school	0.57	(0.40, 0.81)	**0.002**
Religion is important or very important	1.39	(0.95, 2.03)	0.091
Spent 5 years or longer in the U.S.	0.85	(0.58, 1.24)	0.389
Male gender	1.13	(0.81, 1.58)	0.465
Age (continuous)	0.99	(0.98, 1.01)	0.387
Knows someone living or thought to be living with HIV	0.51	(0.24, 1.06)	0.072
**People living or thought to be living with HIV lose respect or standing**			
Cohort / Campaign Exposure			
Post-unexposed	1.28	(0.90, 1.84)	0.172
Post-exposed	1.07	(0.71, 1.61)	0.747
Completed secondary school	0.74	(0.54, 1.01)	0.058
Religion is important or very important	1.00	(0.71, 1.42)	0.985
Spent 5 years or longer in the U.S.	0.97	(0.68, 1.38)	0.860
Male gender	1.33	(0.97, 1.82)	0.072
Age (continuous)	1.00	(0.98, 1.02)	0.988
Knows someone living or thought to be living with HIV	0.93	(0.51, 1.69)	0.805
**People get HIV because they engage in irresponsible behavior**			
Cohort / Campaign Exposure			
Post-unexposed	1.32	(0.85, 2.04)	0.211
Post-exposed	1.85	(1.08, 3.15)	**0.025**
Completed secondary school	0.71	(0.48, 1.05)	0.084
Religion is important or very important	1.61	(1.07, 2.42)	**0.022**
Spent 5 years or longer in the U.S.	0.51	(0.33, 0.80)	**0.004**
Male gender	1.62	(1.09, 2.39)	**0.016**
Age (continuous)	1.02	(1.00, 1.04)	0.053
Knows someone living or thought to be living with HIV	0.43	(0.22, 0.84)	**0.013**
**Four or more stigma beliefs**			
Cohort / Campaign Exposure			
Post-unexposed	1.62	(1.08, 2.43)	**0.020**
Post-exposed	1.76	(1.12, 2.76)	**0.015**
Completed secondary school	0.53	(0.37, 0.77)	**<0.001**
Religion is important or very important	1.60	(1.08, 2.39)	**0.020**
Spent 5 years or longer in the U.S.	0.80	(0.54, 1.19)	0.282
Male gender	1.66	(1.17, 2.36)	**0.005**
Age (continuous)	1.00	(0.99, 1.02)	0.635
Knows someone living or thought to be living with HIV	0.48	(0.22, 1.05)	0.068

^a^ AOR modelling effects of exposure to the *Solo Se Vive Una Vez* social marketing campaign on agreement with stigma belief items among exposed (n = 139) and unexposed (n = 218) post-*Vive* survey respondents, with pre-*Vive* survey respondents (n = 312) as the comparison group for the cohort/exposure effects. AOR models control for statistically significant sociodemographic differences between pre- and post-*Vive* survey populations (education, importance of religion, and time spent in the U.S.) as well as for factors typically associated with stigma from the literature (gender, age, knowing PLHA).

**Table 4:** Effects of cohort and *Vive*-campaign exposure on HIV testing behaviors.

	AOR^a^	(95% CI)	p-value
**Ever tested for HIV**			
Cohort / Campaign Exposure			
Post-unexposed	0.97	(0.66, 1.43)	0.867
Post-exposed	2.25	(1.35, 3.77)	**0.002**
Completed secondary school	0.76	(0.53, 1.08)	0.129
Religion is important or very important	1.17	(0.80, 1.72)	0.419
Spent 5 years or longer in the U.S.	2.61	(1.77, 3.85)	**<0.001**
Male gender	0.44	(0.31, 0.62)	**<0.001**
Age (continuous)	1.00	(0.98, 1.02)	0.867
Knows someone living or thought to be living with HIV	2.02	(0.95, 4.29)	0.066
**Tested for HIV in the past year**			
Cohort / Campaign Exposure			
Post-unexposed	0.55	(0.35, 0.87)	**0.010**
Post-exposed	1.25	(0.79, 1.99)	0.343
Completed secondary school	0.96	(0.65, 1.40)	0.818
Religion is important or very important	0.79	(0.52, 1.21)	0.281
Spent 5 years or longer in the U.S.	0.85	(0.55, 1.31)	0.457
Male gender	1.02	(0.70, 1.50)	0.909
Age (continuous)	1.00	(0.98, 1.02)	0.837
Knows someone living or thought to be living with HIV	1.03	(0.52, 2.05)	0.929

^a^ AOR modelling effects of exposure to the *Solo Se Vive Una Vez* social marketing campaign on HIV testing behaviors among exposed (n = 139) and unexposed (n = 218) post-*Vive* survey respondents, with pre-*Vive* survey respondents (n = 312) as the comparison group for the cohort/exposure effects. AOR models control for statistically significant sociodemographic differences between pre- and post-*Vive* survey populations (education, importance of religion, and time spent in the U.S.) as well as for factors typically associated with stigma from the literature (gender, age, knowing PLHA).

In the adjusted models, a statistically significant increase in agreement with three of the stigma items is observed among those who report that religion is important or very important to them. Respondents for whom religion is important or very important had an increased odds of 1.65 of fearing contracting HIV from the saliva of PLHA (p = 0.006), increased odds of 1.95 of believing that children living with HIV should not be able to attend school with children who are HIV negative (p < 0.001), and an increased odds of 1.61 of agreeing that people get HIV because they engage in irresponsible behavior (p = 0.022). Overall, respondents for whom religion is important or very important had an increased odds of 1.60 of endorsing four or more stigma items (p = 0.02). Males were also significantly more likely to endorse four or more stigma items (AOR = 1.66, 95% CI (1.17, 2.36), p = 0.005).

Exposure to the *Vive* campaign was strongly associated with ever testing for HIV (AOR = 2.25, 95% CI (1.35, 3.77), p = 0.002), but testing in the past year was not significant. Conversely, there was steep and statistically significant reduction in likelihood of recent testing pre-to-post-*Vive* among those unexposed to the campaign (AOR = 0.55, 95% CI (0.35, 0.87), p = 0.01).

## Discussion

*Sólo Se Vive Una Vez*, a multilevel social marketing campaign that included messages addressing the most common HIV-related stigmatizing beliefs and barriers to testing as guided by prior research, was implemented in Baltimore, Maryland over a 6-month period. This study aimed to examine current HIV-related stigma beliefs and HIV testing behaviors in Baltimore, differences in HIV-related stigma beliefs and HIV testing behaviors pre- and post-*Vive* implementation, and differences in HIV-related stigma beliefs and HIV testing behaviors among those exposed to the *Vive* campaign and those not exposed. Respondents to the post-*Vive* survey continued to hold high levels of stigma beliefs, and compared to the pre-*Vive* survey sample, were more likely to hold four or more stigmatizing beliefs (from the six survey items). This pro-stigma trend was less pronounced in the post-*Vive*-exposed respondents, suggesting the campaign may have blunted pro-stigma temporal trends, but was insufficient to negate them. Survey respondents who were exposed to the campaign, however, had an increased odds of 2.25 for reporting ever having been tested for HIV, versus more consistent testing pre-to-post among the unexposed. The unexposed saw a steep and statistically significant decline in recent testing (within the past year) while the opposite trend—a slight, but not statistically significant, increase in recent testing–is observed for the exposed.

Findings from the current survey and the broader *Vive* evaluation demonstrate that the campaign reached a considerable fraction of the Latino community and promoted HIV testing among this group [27, 28]. However, respondents in the post-*Vive* campaign period showed more stigmatizing beliefs than those surveyed pre-*Vive*. The increase in stigmatizing beliefs does not appear to be attributable to the campaign, as pro-stigma trends were observed for both those who were exposed to the campaign versus not. One exception is the blame item, which the campaign may have unintentionally amplified. High levels of HIV-related stigma have been found in Latin American countries [33–35], and immigrants often bring these beliefs with them as they settle in the U.S. Additional research is warranted to determine the role of the messaging of the campaign in influencing beliefs, the alignment of these messages with the beliefs of newly arriving immigrants, and the role of the changing social context in the effectiveness of HIV-related stigma messaging.

Notably, the pre-*Vive* survey occurred prior to the 2016 U.S Presidential election. The Trump presidential campaign and the subsequent actions of the Trump Administration have had numerous detrimental effects on Latino immigrant communities [36–40]. This social context, which increased anti-immigrant sentiment across the U.S. [37, 41], may be an important consideration for understanding stigma within Latino immigrant communities. Stigmatization is a social process linked to the actions of groups of people within specific contexts of culture, power, and inequality [17]. Research on stigma [18, 42] and social identity theory [43] suggest that membership within a stigmatized group, in this case Latino immigrant communities during a time of heightened anti-immigrant sentiment and structural policies increasingly fostering exclusion, can negatively impact a person’s sense of wellbeing and self-esteem. This relationship is complex and situational [44], but when stigmatization negatively impacts self-esteem, people often engage in strategies to overcome this. Stigmatizing others may be one such strategy as it can produce an enhanced sense of wellbeing and improve self-esteem through downward comparison [45] and may provide the perception of control [46]. Members of stigmatized groups have been found to engage in downward comparisons or stigmatization of others within their group as a strategy for improving their own sense of wellbeing, self-esteem, and control [47–49]. Thus, it is possible that the increased stigma observed post-*Vive* may be influenced by the social context of increased anti-immigrant sentiment. The current anti-immigrant landscape certainly does not fully explain HIV-related stigma within Baltimore’s Latino immigrant community; however, in attempting to combat it, this social context may necessitate additional strategies.

Our findings highlight the need for continued efforts within religious and community institutions as a critical component of HIV-related stigma reduction strategies. Among the post-*Vive* survey respondents, those for whom religion was important or very important had an increased odds of 1.6 of holding four or more stigmatizing beliefs (out of six possible). Additionally, these respondents were more likely to blame people living with HIV, believing that irresponsible behaviors lead to infection. Working with non-governmental partners, including faith leaders and institutions, to increase HIV testing and reduce HIV-related stigma is identified in the National HIV/AIDS Strategy as a necessary component to reducing HIV [21]. Religious leaders often have the credibility to mobilize resources to disseminate information, and church-based interventions have shown promise in reducing HIV stigma and promoting HIV testing in Latinos [49–51]. Additionally, immigrant community institutions may be important in shifting stigmatizing beliefs in general and in relation to religion. Soon after arrival in a host location, immigrants create community institutions, such as immigrant community centers and churches. These provide the immigrants with practical, social, and emotional support as they navigate the challenges of life in their new location, while also reproducing various cultural and lifestyle elements from the immigrants’ countries of origin [52]. As such, their involvement in efforts aiming to reduce HIV-related stigma may alter perceptions of the appropriateness of these efforts, influence the beliefs of immigrants who do not attend religious institutions, and increase receptivity to efforts aiming to reduce HIV-related stigma within religious institutions [53].

Despite the significance of our findings, there are several limitations that should be noted. The cross-sectional design limits the ability to make causal associations. Because the study was primarily conducted in street venues, we selected previously validated HIV-related stigma items to develop a short questionnaire that could feasibly be administered in a brief period of time. We conducted the survey using Qualtrics software with audio formatted for each question and response, similar to ACASI, which has been shown to improve response rates and increased reporting of certain behaviors, but there is always potential for social desirability bias. For this reason, the survey did not include measures of enacted stigma, such as asking respondents if they themselves engage in stigmatizing behavior towards others. The pre- and post-campaign surveys were implemented four years apart. Although we attempted to minimize this gap, this time was necessary to develop and implement the campaign. Since this population is rapidly growing, the population currently may be very different than that of four years ago. The pre-*Vive* survey was included in research that informed the campaign; however, specific stigma items were not necessarily directly addressed in the final *Vive* messaging. Although education is a critical component of reducing stigma, the use of measures directly related to the campaign limits our ability to fully understand its effectiveness in reducing stigma. Finally, this survey was conducted primarily among Spanish-speaking, foreign-born Latinos living in Baltimore, English proficiency is generally low among Latino immigrants in Baltimore; however, this was not assessed among the survey participants. Our findings may not be generalizable to Latino populations in other areas; however, our results may be relevant to other urban areas with rapidly growing immigrant Latino communities.

Our findings demonstrate the importance of the changing social context in addressing stigma and the need to regularly assess the messaging and reach of campaigns within emerging immigrant communities to ensure their relevance and impact as the population changes. Additionally, given the association of HIV-related stigmatizing beliefs among those for whom religion was important, religious leaders and institutions must remain a critical partner in efforts to address HIV-related stigma and improve HIV-related outcomes. Continued research is warranted to identify effective social marketing strategies for rapidly changing communities as well as to better understand the influence of anti-immigrant environments on HIV stigmatizing beliefs.

## Supporting information

S1 File (DOCX)

S2 File (DTA)
