# Supplementary material for: HIV-related stigma among Spanish-speaking Latinos in an emerging immigrant city following the Solo Se Vive Una Vez social marketing campaign
Source: PLoS One. 2022 Oct 6;17(10):e0274888. doi: 10.1371/journal.pone.0274888 (PMC9536543; doi:10.1371/journal.pone.0274888)
Supplement: S1 File — (DOCX) [file pone.0274888.s001.docx]

**Community Stigma Survey**

**Your completion of this survey will serve as your consent to be in this research study.**

1. What is your age? ____________

2. What is your gender?

- Male
- Female

3. What country are you from?

⭘ Cuba

⭘ Dominican Republic

⭘ El Salvador

⭘ Ecuador

⭘ Guatemala

⭘ Honduras

⭘ Mexico

⭘ Nicaragua

⭘ Panama

⭘ Peru

⭘ United States

⭘ Other _____________

4. How long have you lived in the U.S.?

⭘ Less than 1 year

⭘ 1-2 years

⭘ 2-3 years

⭘ 3-4 years

⭘ 4-5 years

⭘ More than 5 years

5. What is the highest level of education you completed?

⭘ I never went to school

⭘ Primary school (Grades 1-6)

⭘ Some secondary school/high school studies (Grades 7-12)

⭘ High school diploma or the equivalent

⭘ Technical or vocational school

⭘ Some college studies

⭘ College degree

⭘ Graduate degree

6. Are you married?

- Yes
- No

7. What is your religion?

⭘ Catholic

⭘ Evangelical or Protestant Christian

⭘ Jehova’s Witness

⭘ Mormon

⭘ Jewish

⭘ Islam/Muslim

⭘ Orthodox Church

⭘ Other religion- Christian

⭘ Other religion- non Christian

⭘ No religion

[If No religion, skip to Q9]

8. How important is religion to you?

⭘ Not important

⭘ Somewhat important

⭘ Important

⭘ Very important

9. Have you ever been tested for HIV?

⭘ Yes

⭘ No

[If No, skip to Q11]

10. When was your last HIV test?

⭘ Less than one year ago

⭘ More than one year ago

⭘ I don’t know, or I am not sure

11. Do you personally know anyone who is living with HIV?

⭘ Yes

⭘ No

12. I am hesitant to take an HIV test due to fear of people’s reaction if the test is positive for HIV:

⭘ Yes

⭘ No

⭘ Don’t know/Not sure

13. Do you fear that you could contract HIV if you come into contact with the saliva of a person living with HIV?

⭘ Yes

⭘ No

⭘ Don’t know/Not sure

**People feel differently about people living with HIV. We are now going to ask you about issues relevant to HIV and people living with HIV. Using your own opinions, please tell us how strongly you agree or disagree to the following statements.**

14. I would be ashamed if someone in my family had HIV:

⭘ Yes

⭘ No

⭘ It depends

⭘ Don’t know/Not sure

15. Do people living with or thought to be living with HIV lose respect or standing?

⭘ Yes

⭘ No

⭘ It depends

⭘ Don’t know/Not sure

16. Do you think children living with HIV should be able to attend school with children who are HIV negative?

⭘ Yes

⭘ No

⭘ It depends

⭘ Don’t know/Not sure

17. People get HIV because they engage in irresponsible behaviors

⭘ Agree

⭘ Disagree

⭘ It depends

⭘ Don’t know/Not sure

18. Have you previously seen or heard of the ¡Solo Se Vive Una Vez! campaign?

- Yes
- No

[If No, skip to end]

19. Did you consider testing for HIV after seeing the ¡Solo Se Vive Una Vez! campaign?

- Yes, I got tested
- I thought about it, but did not get tested
- No

20. In what ways did you see or hear about the ¡Solo Se Vive Una Vez! campaign? Select all that apply:

- Website
- Facebook
- Billboard
- Poster
- Cell phone app
- Radio
- Community event

**Thank you for your participation. Please hand the tablet to the health worker.**

**Encuesta de la Comunidad**

**Al completar este cuestionario usted está dando su consentimiento para participar en este estudio de investigación.**

1. ¿Cuál es su edad? __________
2. ¿Cuál es su género?

⭘ Masculino (hombre)

⭘ Femenino (mujer)

3. ¿De dónde viene usted?

⭘ Cuba

⭘ República Dominicana

⭘ El Salvador

⭘ Ecuador

⭘ Guatemala

⭘ Honduras

⭘ México

⭘ Nicaragua

⭘ Panama

⭘ Perú

⭘ Estados Unidos

⭘ Otro país _____________

4. ¿Cuánto tiempo lleva viviendo en los Estados Unidos?

⭘ Menos de un año

⭘ Entre 1 a 2 años

⭘ Entre 2 a 3 años

⭘ Entre 3 a 4 años

⭘ Entre 4 a 5 años

⭘ Más de 5 años

5. ¿Cuál es el nivel de educación más alto que ha completado?

⭘ Nunca fui a la escuela

⭘ Escuela primaria (Grado 1 al 6)

⭘ Hice algunos estudios de escuela secundaria (Grado 7-12)

⭘ Terminé la escuela secundaria o su equivalente

⭘ Escuela técnica o vocacional

⭘ Hice algunos estudios universitarios

⭘ Terminé la universidad

⭘ Terminé estudios de post-grado

6. ¿Está casada?

- Si
- No

7. ¿Cuál es su religión?

⭘ Católico

⭘ Evangélico o Protestante Cristiano

⭘ Testigo de Jehová

⭘ Mormón

⭘ Judío

⭘ Islam/Musulmán

⭘ Iglesia Ortodoxa

⭘ Otra Religión- Cristiana

⭘ Otra Religión- no Cristiana

⭘ Ninguna Religión

[If No religion, skip to Q9]

8. ¿Es importante la religión para usted?

⭘ Para nada

⭘ Algo importante

⭘ Importante

⭘ Muy importante

9. ¿Alguna vez se ha hecho la prueba del VIH?

⭘ Sí

⭘ No

[If No, skip to Q11]

10. ¿Hace cuánto tiempo fue su última prueba de VIH?

⭘ Menos de un año

⭘ Más de un año

⭘ No sé, o no estoy seguro

11. ¿Conoce a alguien que esté viviendo con el VIH?

⭘ Sí

⭘ No

12. Estoy reacio a hacerme la prueba de VIH por temor a la reacción de los demás si la prueba sale positiva:

⭘ Sí

⭘ No

⭘ No sé, o no estoy seguro

13. ¿Tiene miedo de contagiarse con VIH si se pone en contacto con la saliva de alguien que vive con el VIH?

⭘ Sí

⭘ No

⭘ No sé, o no estoy seguro

**La gente tiene opiniones diferentes sobre las personas que viven con el VIH. Le vamos a hacer preguntas sobre temas asociados al VIH y a las personas que viven con el VIH. Algunas de las preguntas pedirán su opinión sobre cómo piensa que las personas con VIH son tratadas. Usando sus propias opiniones, por favor díganos si está de acuerdo o no con las siguientes declaraciones**

14. Me sentiría avergonzado si tuviera un pariente con VIH:

⭘ Estoy de acuerdo

⭘ No estoy de acuerdo

⭘ No sé, o no tengo ninguna opinión al respecto

15. ¿Las personas con VIH, o aquellas de quien se dice que tienen VIH, pierden el respeto de los demás y/o su estatus social?

⭘ Sí

⭘ No

⭘ Depende

⭘ No sé, o no estoy seguro

16. ¿Cree que los niños viviendo con VIH deberían poder asistir a la escuela con niños sin VIH?

⭘ Sí

⭘ No

⭘ Depende

⭘ No sé, o no estoy seguro

17. La gente se infecta con el VIH porque se involucran en conductas irresponsables:

⭘ Estoy de acuerdo

⭘ No estoy de acuerdo

⭘ Depende

⭘ No sé, o no tengo ninguna opinión al respecto

18. ¿Alguna vez ha visto o escuchado la campaña ¡Solo Se Vive Una Vez!?

⭘ Si

⭘ No

[If No, skip to end]

19. ¿Consideró el hacerse la prueba del VIH después de ver la campaña ¡Solo se Vive una Vez!?

⭘ Sí, me hice la prueba

⭘ Lo pensé, pero no me hice la prueba

⭘ No

20. ¿De qué manera se enteró o escuchó de la campaña Solo Se Vive una Vez?

Seleccione todas las opciones que aplican:

⭘ Pagina Web

⭘ Facebook

⭘ Anuncio Panorámico

⭘ Poster

⭘ Aplicación en el Celular

⭘ Radio

⭘ Evento de la Comunidad

**Muchas gracias por su participación. Por favor, entregue esta tableta al trabajador de salud.**
